# Antimycotic Activity of Ozonized Oil in Liposome Eye Drops against *Candida* spp.

**DOI:** 10.1167/tvst.9.8.4

**Published:** 2020-07-02

**Authors:** Giuseppe Celenza, Roberto Iorio, Salvatore Cracchiolo, Sabrina Petricca, Ciro Costagliola, Benedetta Cinque, Bernardetta Segatore, Gianfranco Amicosante, Pierangelo Bellio

**Affiliations:** 1 Department of Biotechnological and Applied Clinical Sciences, University of L'Aquila, L'Aquila, Italy; 2 Department of Medicine and Health Sciences “Vincenzo Tiberio”, University of Molise, Campobasso, Italy; 3 Department of Life, Health and Environmental Sciences, University of l'Aquila, L'Aquila, Italy

**Keywords:** eye fungal infections, ozonated oil, collyrium, membrane potential dissipation, Candida, liposome

## Abstract

**Purpose:**

This study aims to investigate the antifungal activity and mechanism of action of ozonized oil eye drops in liposomes (Ozodrop), commercialized as eye lubricant for the treatment of dry eye syndrome and eye inflammation. The activity was tested against four clinical *Candida* species: *C* *albicans,* *C* *glabrata,* *C* *krusei*, and *C* *orthopsilosis*.

**Methods:**

The antifungal activity of the eye drop solution was ascertained by microdilution method in accordance with EUCAST obtaining the minimum inhibitory concentration for Ozodrop. The mechanism of action was further investigated in *C* *albicans* by measuring cell vitality, intracellular reactive oxygen species production, levels of cellular and mitochondrial (∆Ψ_m_) membrane potential, and the extent of membrane lipid peroxidation.

**Results:**

All *Candida* isolates were susceptible to Ozodrop with minimum inhibitory concentration values ranging from 0.195% (v/v) for *C* *glabrata* to 6.25% (v/v) for *C* *orthopsilosis*. After 1 hour of exposure at the minimum inhibitory concentration value about 30% of cells were killed, reaching about 70% at the highest Ozodrop value. After Ozodrop exposure, *C* *albicans* showed cell membrane depolarization, increased levels of lipid peroxidation, depolarized ∆Ψ_m_, and increased reactive oxygen species generation.

**Conclusions:**

The significant increases in reactive oxygen species production cause the accumulation of reactive oxygen species-associated damages leading to progressive Candida cell dysfunction.

**Translational Relevance:**

The antifungal activity of Ozodrop was demonstrated at concentrations several times lower than the concentration that can be retrieved in ocular surface after its application. The antifungal activity of the eye drops Ozodrop would represent an interesting off-label indication for a product basically conceived as an eye lubricant.

## Introduction

Ocular fungal infections are caused by a large number of filamentous fungi, usually found in post-traumatic infections, such as *Aspergillus* spp. and *Fusarium* spp., and yeast, whose most representative organism is *Candida* spp. Fungal infections can involve any part of the ocular surface like cornea (fungal keratitis), conjunctiva (conjunctivitis), eyelids (blepharitis), and lacrimal glands (dacryoadenitis) and can extend to the interior structures of the eye causing fungal endophthalmitis. They are considered one of the most important causes of vision loss in most part of the world.^1^^,^^2^ For instance, fungal keratitis represent almost one-half of the infectious keratitis and the principal cause of loss vision in Asia, especially in tropical and subtropical regions.^3^^,^^4^ Although the most prevalent causative agent of keratomycosis in rural subtropical regions is represented by filamentous fungi,^5^^,^^6^ *Candida* spp. is preponderant in developed and temperate countries^6^ where the incidence of ocular mycosis has dramatically increased in the last decades also as consequence of the increased number of immunoincompetent or immunocompromised patients, for example, acquired secondary immunosuppression or AIDS.^7^^–^^11^

To date, the diagnosis and treatment of ocular fungal infections remains challenging for ophthalmologists and, although fungal keratitis are usually empirically treated with topical administration of antifungal drugs, they scarcely penetrate the internal structures through ocular surface.^12^^–^^14^ The number of drug formulations is restricted; thus, therapeutic options are limited to a few and often expensive drug preparations.^15^^,^^16^ A Cochrane systematic review demonstrated that there is no evidence about the efficacy of the current available topical preparations, most probably owing to the small sample size of existing studies.^17^ Furthermore, the use of antimicrobial agents increases the appearance of reduced susceptibility, which in the worst cases can evolve in open resistance. The use of antiseptic agents as povidone iodine (polyvinylpyrrolidone-iodine) or chlorhexidine is nowadays widely diffused in the field of ophthalmology in the place of antibiotics, during perioperative prophylaxis in eye surgery.^18^ Povidone iodine and chlorhexidine are antiseptic agents commonly used in prophylaxis whose bactericidal effectiveness in decreasing bacterial load on ocular surface has been widely demonstrated.^19^^–^^21^ To overcome challenges and obstacles in the treatment of fungal ocular infections and the issue of emerging worldwide antimicrobial resistance in fungi, as well as in bacteria, alternative agents must be considered.

In the last decades, the use of ozone in clinical practice has increased for the treatment of numerous pathologies,^22^^–^^25^ including skin diseases,^26^^,^^27^ periodontics,^28^^,^^29^ herniated lumbar discs,^30^ musculoskeletal disorders,^31^ diabetic foot ulcers,^32^ and ocular pain and inflammation.^33^ Ozonated oil can be obtained by a reaction of gaseous ozone and double carbon–carbon bonds of unsaturated fatty acids.^34^ The preparation of ozonides overcomes the intrinsic instability of gaseous ozone, allowing a better exploitation of the pharmaceutical properties of ozone.

This study aims to investigate the antifungal activity of ozonized oil eye drops in liposomes Ozodrop (FB Vision, Ascoli Piceno, Italy), a protective and lubricant solution for ophthalmic use, composed of liposomal sunflower ozonated oil and hypromellose (hydroxypropyl methylcellulose) against four clinical *Candida* species: *C* *albicans,* *C* *glabrata,* *C* *krusei*, and *C* *orthopsilosis*. The mechanism of action on *C* *albicans* was also examined investigating the ability of Ozodrop to modify plasma and mitochondrial membrane potential, as well as the generation of reactive oxygen species (ROS) and lipid peroxidation products.

## Methods

### Fungal Strains

Clinical isolates of *C* *albicans,* *C* *glabrata,* *C* *krusei*, and *C* *orthopsilosis*, were collected from hospitalized patients at the “San Salvatore” Regional Hospital of l'Aquila, Italy.

### Antifungal Activity by Microdilution Method

The antimycotic effect of ozonized oil eye drops in liposomes (Ozodrop, FB Vision) against the organisms used in this study was determined in accordance with EUCAST guidelines (Edef 7.3.1). Briefly, 2×RPMI 2G medium was prepared dissolving RPMI-1640 with L-glutamine, phenol red in 3-(N-morpholino) propanesulfonic acid at a final concentration of 0.165 M, pH 7.0 supplemented with 4% glucose. NaCl 0.9% solution (was used for the dilution of Ozodrop and expressed as percentage of dilution (%v/v) from the undiluted product. All reagents were from Sigma-Aldrich (St. Louis, MO).

The antifungal activity of Ozodrop was ascertained by microdilution method in 96-well microplates statically incubated for 24 hours at 35°C. Microorganism growth, with an initial inoculum of 0.5 McFarland, was spectrophotometrically quantified at 595 nm by microplate reader iMark (BioRad, Hercules, CA). The minimum inhibitory concentration (MIC) for Ozodrop was defined as the concentration of drug that decreases growth by 80% compared with that of organisms grown in the absence of drug. The half inhibitory concentration was extrapolated for each species from the data obtained from microdilution method by nonlinear regression. Excipients were also tested, and no antifungal activity was recorded. To clarify the mechanism of action of Ozodrop we focused our attention on *C* *albicans* as the model organism.

### Membrane Depolarization in *C* *albicans*

The effect of Ozodrop on fungal plasma membrane of *C* *albicans* was investigated by the potentiometric fluorescence probe 3,3’-dipropylthiacarbocyanine iodide (DiSC_3_(3)) using a 0.5 McFarland suspension of *C* *albicans* cells. The fluorescent probe DiSC_3_(3) was added to a final concentration of 1 × 10^−6^ M and the fluorescence intensity was measured with λ_ex_ = 559 nm and λ_em_ = 575 nm by Perkin Elmer Luminescence Spectrometer LS50B (Perkin Elmer, Milan, Italy). Saponin (Sigma-Aldrich) was used as positive control to a final concentration of 0.05% to reach the maximum of depolarization of *C* *albicans* plasma membrane (*D_max_*). The background fluorescence (*D_0_*) of the untreated cells was also measured. Depolarization of *C* *albicans* induced by Ozodrop was calculated as percentage of total released fluorescence (%*TRF*) using the following equation: %*TRF = ((D_S_* *–* *D_0_)/(D_MAX_* *–* *D_0_))* *×* *100*, where *D_s_* represents fluorescence intensity at several concentrations of Ozodrop ranging from 0.05% (v/v) to 3.13% (v/v) corresponding to a range from 1 × MIC to 1/64 × MIC.

### Cell Viability Assay

Cell viability was evaluated using the MTT (3-[4,5-dimethylthiazole-2-yl]-2,5-diphenyltetrazolium bromide) (Sigma-Aldrich) colorimetric method, measuring the metabolic efficiency of living cells. Aliquots of 100 µL of 5 × 10^6^ *–* 10^7^ CFU/mL of *C* *albicans* suspension were incubated for 1 hour at room temperature, with 100 µL of Ozodrop in a range from 12.5% (v/v) (corresponding with 4 × MIC) to 0.78% (v/v) (1/4 × MIC). After incubation, each well was supplemented with 20 µL of a 6 mM stock solution of MTT. The microplates were incubated for 2 hours at 35°C and tetrazolium salts were dissolved by acidic isopropyl alcohol (Sigma-Aldrich) and optical density was spectrophotometrically quantified at 570 nm by microplate reader iMark (BioRad). Data obtained from viability assay were reported as percentage of killed cells with respect to untreated cells.

### Determination of Lipid Peroxidation

Whole-cell lipid membrane peroxidation in *C* *albicans* cells was colorimetrically evaluated by lipid peroxidation assay kit (MAK085, Sigma-Aldrich) following manufacturer's protocol. Briefly, aliquots of 1 mL of 5 × 10^6^ cells of *C* *albicans* suspension in phosphate-buffered saline (PBS) were incubated for 1 hour at room temperature with two-fold dilution in a range from 12.5% (v/v) (4 × MIC) to 0.78% (v/v) (1/4 × MIC) of Ozodrop. An untreated sample was also prepared as a negative control. An aliquot of 200 µL of each reaction mixture was placed into a 96-well clear flat bottom microplate. Absorbance was measured at 530 nm (A_530_) by microplate reader iMark (BioRad). The amount of the malondialdehyde (MDA) in the samples was determined using a standard curve as indicated by the manufacturer.

### Detection of Intracellular ROS

The level of oxidative stress, in terms of ROS production, was detected by using 2’,7’-dichlorofluorescein diacetate (DCFH_2_-DA) (Molecular Probes, Inc., Eugene, OR) as previously reported.^35^ Aliquots of 1 mL of 5 × 10^6^ – 10^7^ CFU/mL of *C* *albicans* suspension were loaded with DCFH_2_-DA to a final concentration of 10 µM and incubated at 28°C for 30 minutes. Cells were then treated for 1 hour with Ozodrop in a range of from 12.5% (v/v) (4 × MIC) to 0.78% (v/v) (1/4 × MIC). To verify ROS induction, 500 µM H_2_O_2_ was used as a positive control. Cells were collected by centrifugation at 14,000*g* for 60 seconds and resuspended in 1 mL of PBS. This step was repeated twice. An aliquot of 500 µL was analyzed by a Luminescence Spectrometer LS50B at λ_ex_ = 504 nm and λ_em_ = 524 nm.

### Cytofluorimetric Analysis of Mitochondrial Membrane Potential

Determination of ∆Ψ_m_ was carried out using the lipophilic cation 5,5′,6,6′-tetrachloro-1,1′,3,3′-tetraethyl-benzimidazolyl carbocyanine iodide (JC-1; Cayman, Ann Arbor, MI) as previously reported.^36^ Aliquots of 10^6^ CFU/mL were treated with different concentrations of Ozodrop, as described elsewhere in this article for the detection of intracellular ROS. Afterward, each sample was incubated with 5 µM JC-1 for 20 minutes at room temperature in the dark. Then, cells were washed in PBS, collected by centrifugation at 14,000*g* for 1 minute and resuspended in 1 mL of PBS. An aliquot of 500 µL for each sample was analyzed by FACSCalibur flow cytometry equipped with Cell Quest software (Becton Dickinson, San Jose, CA) for data acquisition. Data from 5000 events per sample were collected and the fluorescence intensity shift from red to orange was measured and analyzed in the FL1 channel. A sample treated with 50 µM of carbonyl cyanide 4-(trifluoromethoxy)phenylhydrazone (FCCP; Cayman) was used to verify the maximum depolarization.

### Statistical Analysis and Plot

Statistical analysis and plot were performed by OriginPro 2018 software. Unless otherwise indicated, data reported in this study are intended as the mean ± standard error of at least three independent determinations. Comparisons between multiple groups were performed by a one-way analysis of variance repeated measure test, followed by Dunnett test. A *P* value of less than 0.05 was regarded as statistically significant.

## Results

### Antifungal Activity

**Figure 1.**
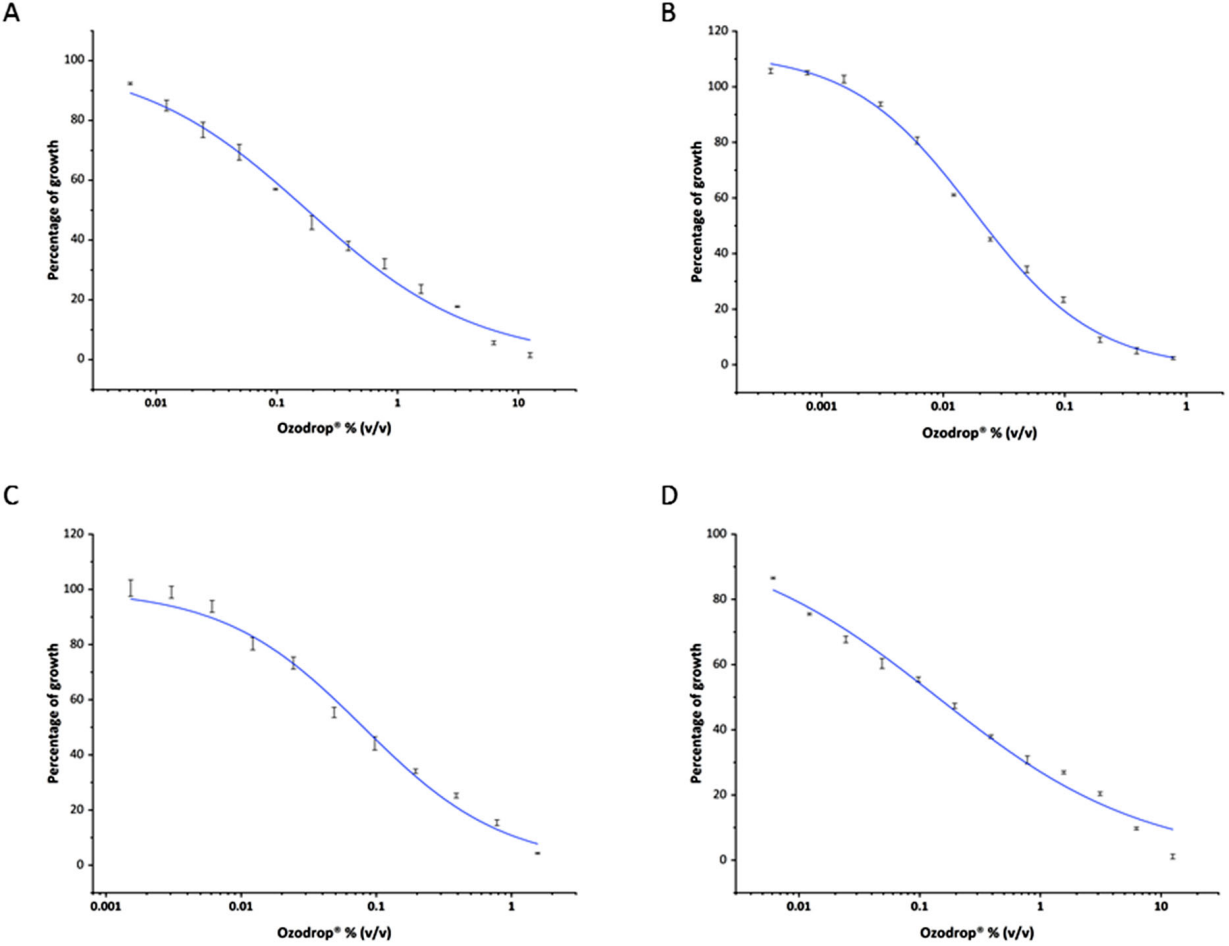
Dose–response plot obtained by optical density at 595 nm used for the determination of the half-inhibitory concentration (*IC*_50_). (A) *C* *albicans*. (B) *C* *glabrata*. (C) *C* *krusei*. (D) *C* *orthopsilosis.* Error is expressed as ± standard error (SEM) of the mean of three independent experiments.

Antifungal activity was preliminarily determined by microdilution method after 24 hours incubation as suggested by EUCAST. As shown in the Table, all *Candida* isolates were susceptible to Ozodrop treatment with MIC values ranging from 0.195% (v/v) for *C* *glabrata* to 6.25% (v/v) for *C* *orthopsilosis*. The half inhibitory concentration was also evaluated by nonlinear regression of optical density assessed at 595 nm (Fig. 1). Half inhibitory concentration values were ranging from about 0.02% (v/v) for *C* *glabrata* to 0.2% (v/v), 10-fold higher, for *C* *albicans* (Table). The mechanism of action was further investigated in *C* *albicans*, which was used as the model organism.

**Table.** Half Inhibitory Concentration (*IC_50_*) and MIC Values of Ozonized Oil in Liposomes (Ozodrop) Evaluated After 48 Hours of Incubation

Organism	*IC_50_* (% v/v)	MIC Value (% v/v)
*C* *albicans*	0.1791 ± 0.0102	3.125
*C* *glabrata*	0.0177 ± 0.0011	0.195
*C krusei*	0.0803 ± 0.0047	0.78
*C* *orthopsilosis*	0.1403 ± 0.0091	6.25

MIC is defined as the concentration (% v/v) of product that reduces growth by 80% compared with the growth of the control.

### Membrane Depolarization

**Figure 2.**
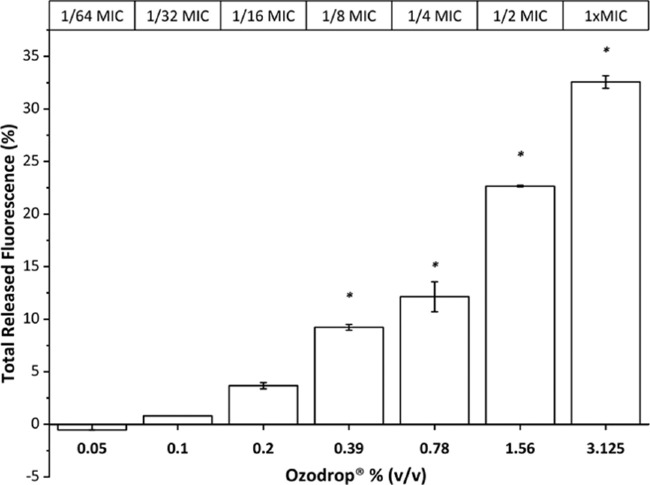
*C* *albicans* membrane depolarization assessed by DiSC_3_(3) released in presence of increased concentrations of Ozodrop. The percentage of the TRF has been calculate as the fluorescence released with respect to the positive control saponin at 0.05% where the maximum of depolarization of C albicans plasma membrane is obtained. Comparison between multiple groups was performed by one-way analysis of variance repeated measures test, followed by Dunnett test. Statistically significance was calculated on raw data (see Supplementary Table S2 and Supplementary Fig. S2) with respect to the intensity of fluorescence released in presence of Ozodrop. Error is expressed as ± standard error (SEM) of the mean of three independent experiments. A *P* value of less than 0.05 (*) was regarded as statistically significant. More detailed information are available in Supplementary Figure S1 and Supplementary Table S1.

Transmembrane potential dissipation induced by Ozodrop was investigated in *C* *albicans* by using the potentiometric fluorescent probe DiSC_3_(3). Membrane depolarization occurs instantaneously after exposure to Ozodrop in a dose-dependent relationship at concentrations ranging from 1/32 of MIC to MIC value corresponding to 0.1% (v/v) and 3.125% (v/v), respectively. At the MIC value, more than 30% of fluorescence release is observed compared with positive control saponin (Fig. 2). A comparison between multiple groups was performed by a one-way analysis of variance repeated measures test, followed by Dunnett test. Statistical significance was calculated on raw data (Supplementary Table S1 and Supplementary Fig. S1) with respect to the intensity of fluorescence released in presence of Ozodrop.

### Cell Viability Assay

**Figure 3.**
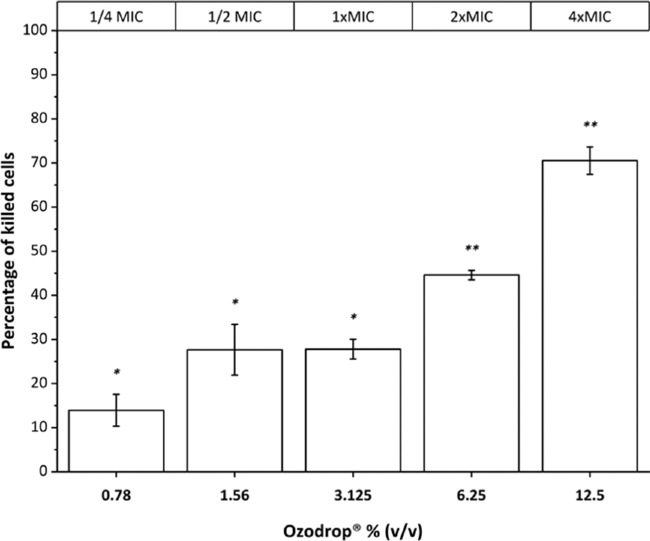
*C* *albicans* mortality after 1-hour incubation assessed by MTT obtained at increasing concentrations of Ozodrop. Comparison between multiple groups was performed by one-way analysis of variance repeated measures test, followed by the Dunnett test. Error is expressed as ± standard error of the mean (SEM) of three independent experiments. Levels of *P* < 0.05 (*) and *P* < 0.0001 (**) were regarded as statistically significant. More detailed information are available in Supplementary Figure S2 and Supplementary Table S2.

Cell viability was assessed by MTT after 1 hour exposure at several Ozodrop concentrations (from ¼ × MIC to 4 × MIC). As shown in Figure 3 at 3.125% (v/v), corresponding with the MIC value, about 30% of cells were killed, reaching about 70% at the highest Ozodrop concentration. Comparison between multiple groups was performed by a one-way analysis of variance repeated measures test, followed by the Dunnett test. Statistical significance was calculated on raw data (see Supplementary Table S2 and Supplementary Fig. S2) with respect to the optical density at 570 nm of the untreated control.

### Ozodrop Increases Levels of Lipid Peroxidation

**Figure 4.**
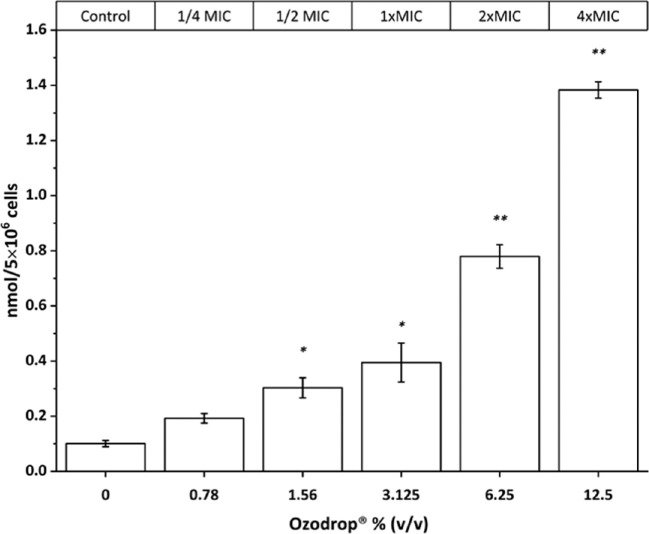
*C* *albicans* membrane lipid peroxidation obtained at increased concentrations of Ozodrop. Comparison between multiple groups was performed by one-way analysis of variance repeated measures test, followed by the Dunnett test. Error is expressed as ± standard error (SEM) of the mean of three independent experiments. A *P* values of less than 0.05 (*) and a *P* value of less than 0.0001 (**) were regarded as statistically significant. More detailed information are available in Supplementary Figure S3, Supplementary Table S3, and Supplementary Table S4.

Lipid peroxidation was determined in *C* *albicans* treated for 1 hour at Ozodrop concentrations ranging from 1/4 × MIC to 4 × MIC (0.78%–12.5% [v/v]) following the generation of MDA and expressed as nmol of MDA produced normalized to the number of cells (5 × 10^6^ CFU). Increasing in lipid peroxidation is observed in a dose-response relationship, as shown in Figure 4. At the MIC value, about 0.4 nmol of MDA per 5 × 10^6^ CFU are produced, and this value reaches about 1.4 nmol at four-fold MIC. Calibration curve was obtained at several concentrations of standard solutions of MDA (see Supplementary Fig. S3, Supplementary Table S3, and Supplementary Table S4).

### Ozodrop Induces Intracellular ROS Production

**Figure 5.**
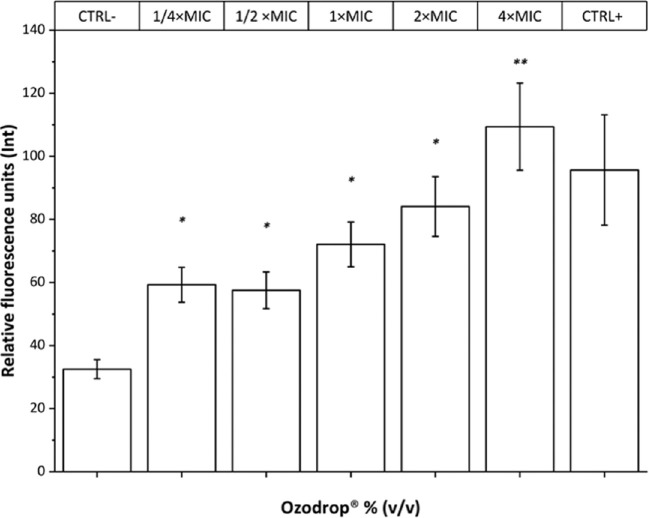
*C* *albicans* ROS generation after 1 hour of incubation obtained at increased concentrations of Ozodrop. CTRL-, negative control; CRTL+, positive control with 500 µM H_2_O_2_. Comparison between multiple groups was performed by analysis of variance one-way repeated measure test, followed by Dunnett test. Error is expressed as ± standard error (SEM) of the mean of three independent experiments. A *P* values of less than 0.05 (*) and a *P* value of less than 0.0001 (**) were regarded as statistically significant. More detailed information are available in Supplementary Table S5.

The fluorescent probe DCFH_2_-DA was used to assess ROS generation upon Ozodrop treatment in a range from 1/4 × MIC to 4 × MIC (0.78%–12.5% [v/v]). Statistically significant enhancement in fluorescence intensity, related to ROS levels generated by Ozodrop, is observed as shown in Figure 5. Hydrogen peroxide was used as positive control. See Supplementary Table S5 for experimental data and statistics.

### Ozodrop Alters ∆Ψ_m_

**Figure 6.**
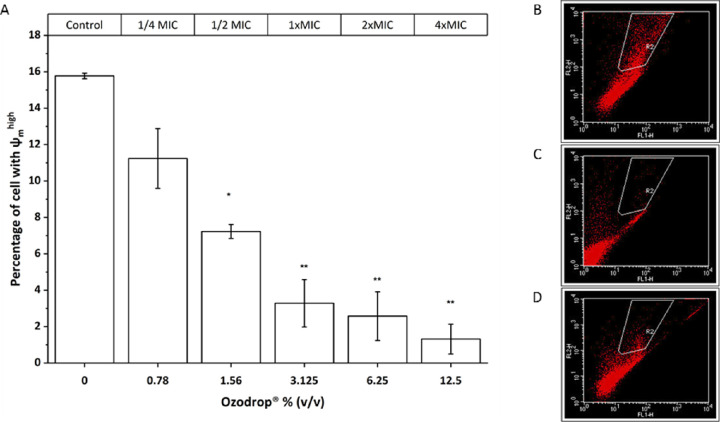
Ozodrop treatment causes mitochondrial membrane potential loss (∆Ψ_m_). ∆Ψ_m_ was measured by means of JC-1 staining. (A) A dose-dependent effect of Ozodrop in *C* *albicans*. The histograms represent the percentage of cells with ∆Ψ_m_^high^ (red fluorescence of JC-1 red in the R2 gate) obtained by flow cytometric analysis. Representative results at 1 hour of incubation for (B) untreated control cells, (C) 50 µM of FCCP used as positive control for the abolishment of ∆Ψ_m_, and (D) 12.5% (v/v) (4 × MIC) of Ozodrop. Comparison between multiple groups was performed by one-way analysis of variance repeated measures test, followed by Dunnett test. Error is expressed as ± standard error (SEM) of the mean of three independent experiments. A *P* values of less than 0.05 (*) and a *P* value of less than 0.0001 (**) were regarded as statistically significant. More detailed information are available in Supplementary Material Table S6.

The overproduction of ROS could suggest mitochondrial dysfunction. Because ∆Ψ_m_ is a key indicator of mitochondrial function, we next examined whether Ozodrop-induced ROS production was associated with changes in ∆Ψ_m_. In this study, mitochondrial activity was measured by flow cytometry using JC-1 staining. As shown in Figure 6, the percentage of cells with ∆Ψ_m_^high^ was significantly decreased in ozone-exposed groups compared with controls, and the effect was dose dependent. These results clearly indicate that treatment leads to alterations in mitochondrial function. See Supplementary Table S6 for experimental data and statistics.

## Discussion

In the last two decades, the use of ozone as a therapeutic option for the treatment of several pathologies has increased, basically owing to the absence of toxicity at the effective therapeutic dosage.^22^ Ozone is commonly used as a sanitizer for several and different purposes in a wide number of fields. The antimicrobial, antifungal, and antiviral actions are well-known, as well as its ability to prevent the development of antimicrobial resistance and allergic reaction, because the product of reaction is molecular oxygen. The use of ozonized oils to treat external urogenital or wound infections is especially encouraged when the infection is sustained by multidrug-resistant strains.^22^ In ophthalmology, ocular infections, to a great extent those sustained by fungi, are difficult to treat. Specifically, fungal keratitis, which are often induced by filamentous fungi (*Aspergillus*, *Fusarium*) or yeast-like fungi (*C* *albicans* and other *Candida* species), represent the principal cause of vision loss in Asia and other tropical and subtropical countries.^3^^,^^4^ Specifically, they are more dangerous and devastating than bacterial keratitis and their diagnostic path and treatment is challenging. Moreover, *Candida albicans* keratitis are frequently associate with systemic illness or complicated chronic ocular diseases.

During exposure to ozone, oxidation of bacterial and fungal membrane phospholipids and lipoproteins leads to alterations of the stability and integrity of cellular membrane.^24^^,^^25^^,^^37^ This exposure induces oxidation of cytosolic components, which is followed by bacterial and fungal death. However, gaseous ozone is unfortunately unstable and, consequently, difficult to use. Ozonated oil is the product of reaction of gaseous ozone and unsaturated fatty acids, which leads to the formation of ozonated cyclic species, such as 1,2,4-trioxolane ring, the active portion of the ozonized fatty acid molecule.^34^^,^^38^ So far, the demonstrated antimicrobial activity of ozonized oils has been exploited in several fields of application.^29^^,^^39^^–^^43^

In this study, we demonstrated the antifungal activity of the ozonized sunflower oil eye drops in liposome against four species of *Candida*. The antimicrobial activity was demonstrated at concentrations several times lower than those of the undiluted product retrieved in ocular surface after the topical of Ozodrop.^44^ Transmembrane potential dissipation, measured by the potentiometric fluorescent probe DiSC_3_(3), can be observed instantaneously after exposure to Ozodrop (Fig. 2). The dissipation of the cell membrane potential is the direct consequence of the of the membrane structure alteration, presumably owing to membrane lipid peroxidation, as demonstrated in Figure 4 in whole cell assay. The attack of the ozonized oil, specifically of the trioxolane moiety, causes peroxidation of the membrane unsaturated lipids, leading to deformation in terms of structure and functionality of the plasma membrane.^45^ In addition, it is known that about 70% of the plasma membrane of *C* *albicans* are made of polyunsaturated fatty acids. Mitochondria are important for energy production, the modulation of calcium signaling, the induction of cell death, as well as the regulation of cellular redox status. It is important to note that mitochondrial membranes are also characterized by a high lipid content. In this regard, the mechanism underlying Ozodrop-induced cytotoxic effects may also involve alterations in mitochondrial membrane fluidity. The generation of lipid peroxidation products may contribute to modify the activity of that organelle, perhaps through inhibition of the respiratory chain. In line with this speculation, the transmembrane potential dissipation observed after exposure to Ozodrop occurs in association with a marked decrease in ∆Ψ_m_ (Fig. 6). Mitochondria are considered the primary source of ROS in the cell. Considering the important relation between ∆Ψ_m_ and the rate of ROS formation, we measured intracellular levels of ROS upon Ozodrop treatment (Fig. 5). Our data clearly indicate that treatment stimulated generation of oxidative stress in *C* *albicans*. This result suggests that ROS production may act as an important factor contributing to Ozodrop cytotoxicity. For instance, significant increases in the levels of ROS production cause the accumulation of ROS-associated damages in lipids, proteins, and DNA leading to progressive cell dysfunction.

## Conclusions

ROS accumulation may play an important role in the mechanism of Ozodrop-induced toxicity in *C* *albicans*. Moreover, we emphasize the potential contribution of mitochondrial activity in mediating oxidative stress. The remarkable in vitro antifungal activity demonstrated in this study sounds especially interesting if we consider that Ozodrop is commercialized as simple eye lubricant, with indications for the treatment of dry eye syndrome, inflammation caused by environmental stress or contact lens, and instability of the tear film. Owing to the lack of any adverse effect, the antifungal activity of Ozodrop would represent an interesting off-label indication.

## Supplementary Material

Supplement 1
