# Supplementary material for: Antimycotic Activity of Ozonized Oil in Liposome Eye Drops against Candida spp
Source: Transl Vis Sci Technol. 2020 Jul 2;9(8):4. doi: 10.1167/tvst.9.8.4 (PMC7422790; doi:10.1167/tvst.9.8.4)
Supplement: Supplement 1 [file tvst-9-8-4_s001.pdf]

Supplementary material

TITLE

Antimycotic activity of ozonized oil in liposome eye drops against *Candida* spp.

AUTHORS

Giuseppe Celenza[1*], Roberto Iorio[1*], Salvatore Cracchiolo[1], Sabrina Petricca[1], Ciro Costagliola[2], Benedetta Cinque[3], Bernardetta Segatore[1], Gianfranco Amicosante[1], Pierangelo Bellio[1].

[1]Department of Biotechnological and Applied Clinical Sciences, University of L'Aquila, Via Vetoio, 1, 67100, L'Aquila, Italy.

[2]Department of Medicine and Health Sciences "Vincenzo Tiberio", University of Molise, Via Francesco De Sanctis 1, 86100, Campobasso, Italy.

[3]Department of Life, Health and Environmental Sciences, University of l'Aquila, Via Vetoio, 1, 67100, L'Aquila, Italy.

Corresponding author:
Giuseppe Celenza
Department of Biotechnological and Applied Clinical Sciences
University of L'Aquila
Via Vetoio, 1
67100 L'Aquila
Italy
giuseppe.celenza@univaq.it
tel +390862433444

Footnote

*authors equally contributed to this study

MEMBRANE DEPOLARIZATION IN *C. albicans*

Table S1. Intensity of fluorescence D_0, background; D_s, released fluorescence at several concentrations of Ozodrop®; D_{max}, maximum of depolarization of *C. albicans* plasma membrane in presence of saponin 0.05%. Fluorescence intensity was measured with λ_{ex} = 559 nm and λ_{em} = 575 nm by Perkin Elmer Luminescence Spectrometer LS50B (Perkin Elmer, Italy) Comparisons between multiple groups were performed by ANOVA One-way repeated measure test on raw data, followed by Dunnett test with respect to D_0. Error is expressed as ± standard error (SEM) of the mean of three independent experiments.

	D_0		D_s			D_{max}		
**Ozodrop® %(v/v)**	**Mean**	**±SE of mean**	**Mean**	**±SE of mean**	***p-value***	**Mean**	**±SE of mean**	***p-value***
0.05	50.73	0.63	48.77	0.90	0.80097	358.04	4.87	1.56634E-7
0.10	52.80	2.37	56.12	1.82	0.84611	372.64	10.31	2.04369E-6
0.20	52.58	1.26	63.78	1.31	0.05863	358.10	5.23	1.93309E-7
0.39	54.65	1.27	82.86	0.91	4.38938E-4	360.32	3.75	3.33129E-8
0.78	50.56	1.07	84.19	5.05	0.02962	327.67	11.14	9.62392E-6
1.56	54.58	1.10	119.83	5.12	8.31708E-4	353.48	9.64	2.0071E-6
3.125	59.85	2.02	162.64	8.38	2.57142E-4	380.87	11.79	2.78997E-6

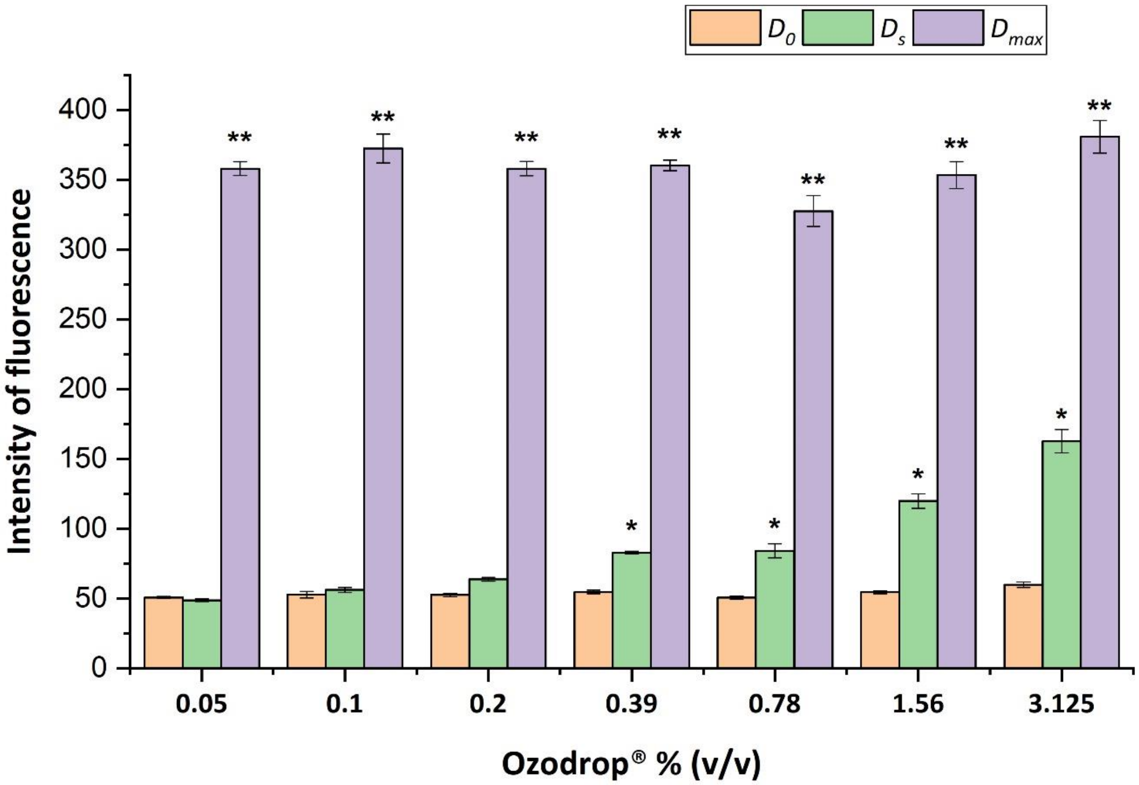

Figure S1. Intensity of released fluorescence in presence of several Ozodrop® dilutions**.** *, p<0.05; **p<0.0001. More detailed information are reported above in Table S2.

CELL VIABILITY ASSAY

Table S2. Cell viability was evaluated using the MTT (3-[4,5-dimethylthiazole-2-yl]-2,5-diphenyltetrazolium bromide. Data obtained from viability assay were reported as percentage of killed cells with respect to untreated cells. Comparisons between multiple groups were performed by ANOVA One-way repeated measure test on raw data, followed by Dunnett test with respect to untreated cells. Error is expressed as ± standard error (SEM) of the mean of three independent experiments.

	Raw data (O.D. at 570 nm)			% of killed cells	
**Ozodrop® %(v/v)**	**Mean**	**±SE of mean**	***p-value***	**Mean**	**±SE of mean**
0	0.84	0.02		0.00	0.00
0.78	0.72	0.03	0.0454	13.93	3.60
1.56	0.60	0.04	4.72E-04	27.66	5.75
3.125	0.60	0.01	4.64E-04	27.79	2.23
6.25	0.46	0.01	6.55E-06	44.59	1.07
12.5	0.25	0.03	4.40E-11	70.53	3.10

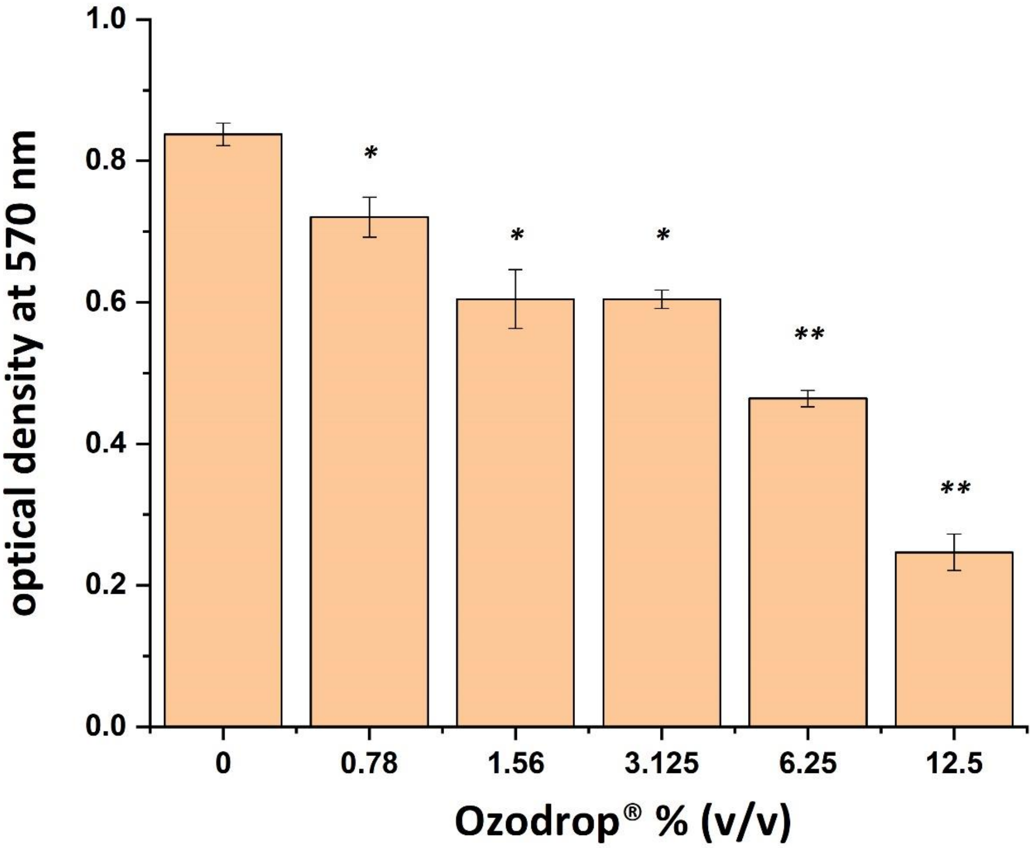

Figure S2. Raw data from optical density at 570 nm. *, $p<0.05$; **$p<0.0001$. More detailed information are reported above in Table S3.

DETERMINATION OF LIPID PEROXIDATION

Whole-cell lipid membrane peroxidation in *C. albicans* cells was colorimetrically evaluated by lipid peroxidation assay kit (MAK085, Sigma-Aldrich, St. Louis, MO, USA) following manufacturer's protocol. Absorbance was measured at 530 nm (A_{530}) by microplate reader iMark, BioRad. The amount of the malondialdehyde (MDA) in the samples was determined using a standard curve as indicated by the manufacturer. Calibration curve was obtained by measuring the absorbance at 530 nm of standard solutions of malondialdehyde in a range from 0 to 20 nmol.

Table S3. Standard solutions of malondialdehyde used for the determination of the calibration curve.

Malondialdehyde (nmol)	Replicates (ABS at 530 nm)			Mean (ABS at 530 nm)	±SE of mean
0.00	0.04	0.053	0.07	0.05	0.00953
4.00	0.25	0.256	0.26	0.26	0.00463
8.00	0.44	0.462	0.46	0.45	0.00689
12.00	0.64	0.637	0.64	0.64	0.00203
16.00	0.82	0.845	0.85	0.84	0.0104
20.00	1.13	1.110	1.110	1.115	0.00709

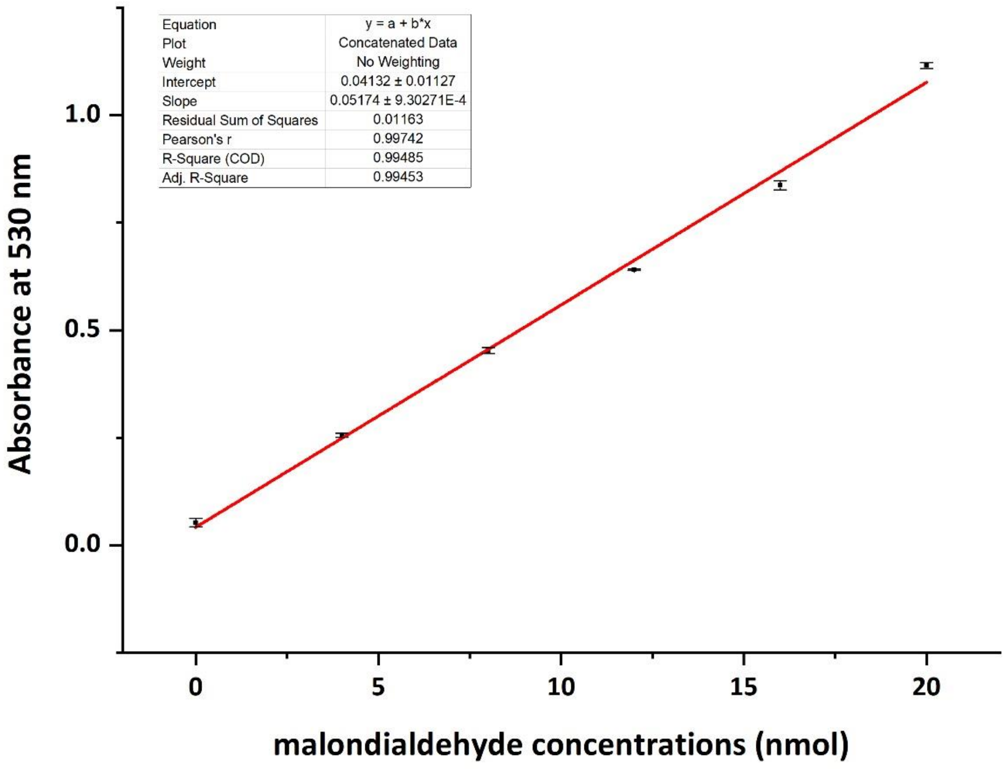

Figure S3. Calibration curve of malondialdehyde obtained by linear regression of concatenated data. Error is expressed as ± standard error (SEM) of the mean of three independent experiments.

Table S4. Whole-cell lipid membrane peroxidation in *C. albicans* cells was colorimetrically evaluated by lipid peroxidation assay kit (MAK085, Sigma-Aldrich, St. Louis, MO, USA) following manufacturer's protocol. Absorbance was measured at 530 nm (A_{530}) by microplate reader iMark, BioRad. Comparisons between multiple groups were performed by ANOVA One-way repeated measure test on raw data, followed by Dunnett test with respect to untreated cells. Error is expressed as ± standard error (SEM) of the mean of three independent experiments.

Ozodrop® %(v/v)	Mean	±SE of mean	*p-value*
0.00	0.10	0.01	
0.78	0.19	0.02	0.33201
1.56	0.30	0.04	0.01167
3.13	0.39	0.07	8.58E-04
6.25	0.78	0.04	1.20E-08
12.50	1.38	0.03	0

OZODROP® INDUCES INTRACELLULAR ROS PRODUCTION

Table S5. The fluorescent probe DCFH2-DA was used to assess ROS generation upon Ozodrop® treatment in a range from 1/4×MIC to 4×MIC (0.78% – 12.5% (v/v)). Statistically significant enhancement in fluorescence intensity, related to ROS levels generated by Ozodrop®, is observed. Hydrogen peroxide at 500 µM was used as positive control. Comparisons between multiple groups were performed by ANOVA One-way repeated measure test on raw data, followed by Dunnett test with respect to untreated cells. Error is expressed as ± standard error (SEM) of the mean of three independent experiments.

Ozodrop® %(v/v)	Mean	±SE of mean	*p-value*
0.00	32.52	3.01	
0.78	59.30	5.53	0.01531
1.56	57.52	5.82	0.02274
3.13	72.07	7.08	0.00107
6.25	84.10	9.50	1.14E-04
12.50	109.42	13.78	1.37E-06
H_2O_2 500 µM	95.67	17.49	

CYTOFLUORIMETRIC ANALYSIS OF MITOCHONDRIAL MEMBRANE POTENTIAL

Table S6. Determination of $\Delta\Psi_m$ was carried out using the fluorescent probe JC-1. Samples were analysed by FACSCalibur flow cytometry equipped with Cell Quest software (Becton Dickinson, San Jose, CA, USA) for data acquisition. Data from 5,000 events per sample were collected and the fluorescence intensity shift from red to orange was measured and analysed in FL1 channel. A sample treated with 50 µM of Carbonyl cyanide 4-(trifluoromethoxy)phenylhydrazone (FCCP; Cayman, Michigan, USA) was used to verify the maximum depolarization. Comparisons between multiple groups were performed by ANOVA One-way repeated measure test on raw data, followed by Dunnett test with respect to untreated cells. Error is expressed as ± standard error (SEM) of the mean of three independent experiments.

Ozodrop® %(v/v)	Mean	±SE of mean	*p-value*
0.00	15.77	0.15	
0.78	11.24	1.64	0.06991
1.56	7.23	0.39	1.55E-03
3.12	3.28	1.31	5.90E-05
6.25	2.58	1.34	2.97E-05
12.50	1.32	0.82	7.96E-06
